# Absence of pathogenic mitochondrial DNA mutations in mouse brain tumors

**DOI:** 10.1186/1471-2407-5-102

**Published:** 2005-08-16

**Authors:** Michael A Kiebish, Thomas N Seyfried

**Affiliations:** 1 Department of Biology, Boston College, Chestnut Hill, MA 02467 USA

## Abstract

**Background:**

Somatic mutations in the mitochondrial genome occur in numerous tumor types including brain tumors. These mutations are generally found in the hypervariable regions I and II of the displacement loop and unlikely alter mitochondrial function. Two hypervariable regions of mononucleotide repeats occur in the mouse mitochondrial genome, i.e., the origin of replication of the light strand (O_L_) and the Arg tRNA.

**Methods:**

In this study we examined the entire mitochondrial genome in a series of chemically induced brain tumors in the C57BL/6J strain and spontaneous brain tumors in the VM mouse strain. The tumor mtDNA was compared to that of mtDNA in brain mitochondrial populations from the corresponding syngeneic mouse host strain.

**Results:**

Direct sequencing revealed a few homoplasmic base pair insertions, deletions, and substitutions in the tumor cells mainly in regions of mononucleotide repeats. A heteroplasmic mutation in the 16srRNA gene was detected in a spontaneous metastatic VM brain tumor.

**Conclusion:**

None of the mutations were considered pathogenic, indicating that mtDNA somatic mutations do not likely contribute to the initiation or progression of these diverse mouse brain tumors.

## Background

Mitochondrial DNA (mtDNA) is essential for the production of subunits for the electron transport chain, since it encodes peptides for Complex I, III, IV, and V. The mitochondrial genome consists of a 16.5 kb piece of circular DNA encoding 37 genes on the L and H strands. These include 22 tRNAs, 2 rRNAs, and 13 respiratory chain subunits [1]. The mitochondrial genetic code varies slightly from the nuclear genetic code and the mutation rate of the mitochondrial genome is 10 fold higher than that of the nuclear genome [2-4]. This higher mutation rate may be due in part to a lack of protective histones, reduced fidelity of polymerase γ, or to the localization of the mitochondrial genome to the inner mitochondrial membrane near damaging reactive oxygen species [5-9]. mtDNA mutations may also contribute to a decrease in metabolic efficiency that enhances aging, promotes neurodegeneration, and is conducive to carcinogenesis [10-12].

mtDNA mutations have been studied in various types of cancers, including breast [13], bladder, lung, head and neck [14], gastric [15], esophageal [16], pancreatic [17], and colon [18]. The majority of the mutations in the mitochondrial genome of human tumors were found in the hypervariable regions of the displacement loop (D-loop) [19-21]. Alterations in these hypervariable regions are unlikely to alter mitochondrial function or produce a diseased phenotype [22]. These hypervariable regions correspond to the O_L _and Arg tRNA in mouse mtDNA [23]. Otto Warburg originally emphasized that the high glycolytic rate of tumors resulted from diminished or disturbed respiration [24,25]. Recent studies suggest that mtDNA mutations may contribute to the respiratory defects in cancer [18]. Furthermore, studies of human tumors have demonstrated an association between mtDNA somatic mutations and tumor progression [26-28].

Few studies have evaluated mtDNA mutations in brain cancer. Mitochondrial genome instability was found in the hypervariable regions in the D-loop of human gliomas, meningiomas, schwannomas, gliomatosis cerebri, glioblastomas, astrocytomas, and neurofibromas [29-33]. Other studies of the mitochondrial genome focused on mtDNA copy number in brain tumors [34]. In the only study examining the entire mitochondrial genome, Wong *et al*. found that the majority of mtDNA somatic mutations in medulloblastomas existed in regions of mononucleotide repeats, rather than in genes encoding proteins for the respiratory chain complexes [35]. A recent study in mouse tumorigenic fibroblasts, suggest that nuclear mutations rather than mtDNA mutations contribute to the tumor phenotype [36]. It is not yet clear if mtDNA mutations contribute to brain tumor progression.

In contrast to most tissues where mitochondrial morphology and function is relatively homogeneous, mitochondria in brain are heterogeneous and exist as non-synaptic free mitochondria (FM), synaptic light mitochondria (LM), and synaptic heavy mitochondria (HM) [37,38]. This morphological heterogeneity is thought to reflect the metabolic heterogeneity of mitochondria within and between the various neuronal and non-neuronal (glial) cell populations [39]. Besides morphological heterogeneity, genetic heterogeneity in mtDNA has been reported in normal human brain tissue [40]. In this study, we sequenced the entire mitochondrial genome in a series of chemically induced and spontaneous mouse brain tumors that differ in metastasis, malignancy, and vascularity. To our knowledge, no prior studies have compared the sequence of the entire mitochondrial genome in brain tumor cell lines with that of the syngeneic brain tissue. Although we found several mutations, none of these were considered pathogenic.

## Methods

### Mice and brain tumors

The inbred VM and C57BL/6J (B6) mouse strains were obtained originally from Professor H Fraser, University of Edinburgh, and from the Jackson Laboratory, Bar Harbor, ME, respectively. All mice used in the study were propagated in the animal care facility at Boston College using animal husbandry conditions described previously [41]. All animal-use procedures were in accordance with the NIH Guide for the Care and Use of Laboratory Animals and were approved by the Institutional Animal Care Committee.

The EPEN and CT-2A brain tumors were produced originally from implantation of 20-methylcholanthrene into the brains of B6 mice as previously described [42,43]. The EPEN tumor arose in the cerebral ventricle and was characterized as an ependymoblastoma, whereas the CT-2A tumor arose in the cerebral cortex and was characterized as a poorly differentiated astrocytoma [42,44]. The CT-2A tumor contains complex gangliosides, is highly angiogenic *in vivo*, and grows *in vitro *as a fusiform monolayer [42,45,46]. In contrast, the EPEN tumor expresses only the simple ganglioside GM3, is less angiogenic *in vivo*, and grows *in vitro *as cuboidal cell islands [41,42,46-49]. The nonmetastatic (VM-NM) and the metastatic (VM-M) tumors arose spontaneously in the brains of VM mice as previously described for other VM brain tumors [50,51].

### Tumor cell culture

Cultured cell lines were prepared from each tumor as we previously described [52,53]. The number of passages for CT-2A and EPEN cell lines were greater than 40, where as VM-NM and VM-M were passaged 9 and 11 times, respectively. The VM-M cell line maintained metastatic capacity when implanted into VM mice. The tumor cells were grown in Dulbecco's modified Eagle medium (DMEM) supplemented with 10% heat-inactivated fetal bovine serum (FBS) in a humidified atmosphere containing 95% air and 5% CO_2 _at 37°C. Upon reaching confluency, the cells were trypsinized and collected.

### Mitochondrial isolation from brain tissue and cultured tumor cells

Mitochondria were isolated from VM and B6 cerebral cortex according to previously described procedures [38,54]. Cerebral cortex mitochondria were separated into three populations following ficoll gradient centrifugation. These populations included non-synaptic free mitochondria (FM), synaptic light mitochondria (LM), and synaptic heavy mitochondria (HM). Mitochondria were isolated from the tumor cell lines as a single enriched mitochondrial fraction as previously described [54,55]. Mitochondria were isolated in duplicate independent samples from each of the brain tumor cell lines for sequence comparison.

### Isolation and amplification of mouse mtDNA

mtDNA from cerebral cortex and cultured tumor cells was isolated using the DNeasy Tissue Kit (QIAGEN, Valencia, CA, USA) and was amplified in 12 overlapping segments covering the entire mitochondrial genome. PCR reactions were performed in a volume of 50 μl using the Eppendorf Master Taq system (Brinkmann Eppendorf, Westbury, NY). Primers used for mtDNA amplification are given in Table 1. PCR conditions for all primer pairs consisted of an initial denaturation at 94°C for 120 s followed by 34 cycles of 94°C for 60 s, an annealing temperature of 57–66°C for 35 s and 72°C for extension at 105 s. A final extension was for 5 min. Optimal annealing temperatures were determined by performing gradient PCR on each of the primer sets.

### mtDNA sequencing

Amplified regions of mtDNA were purified using a PCR purification kit (MO BIO Laboratories, Solana Beach, CA) and were then visualized by 1% agarose gel electrophoresis. DNA concentration was estimated using a Low DNA Mass Ladder (Invitrogen, Carlsbad, CA) by gel electrophoresis. Approximately 25 fmol of the purified PCR product was used in the sequencing reaction following manufacturer's instructions for the CEQ DTCS kit (Beckman Coulter, Fullerton, CA). The amount of DNA used varied depending on the size of the PCR fragment. The primers in Table 1, together with 75 nested primers, were used in the sequencing reactions to obtain double-stranded sequence. Nested primer information can be made available upon request to corresponding investigators.

Sequencing reactions were performed at 96°C for 20 s, 50°C for 20 s, and 60°C for 240 s based on a 32 cycle reaction. Sequencing products were ethanol precipitated in cold 95% ethanol, washed twice with cold 70% ethanol, dried for 20 minutes, and then re-dissolved in sample loading solution provided by the manufacturer (Beckman Coulter, Fullerton, CA). The mtDNA sequence alignments were then compared to those from *Mus Musculus *[GenBank: V00711] [56] and from B6 mice [GenBank: AY172335] [23].

## Results

### Analysis of mtDNA variation in B6 and VM mouse strains

Sequence analysis of the entire mitochondrial genome of the VM and B6 mouse strains revealed a single nucleotide base pair change at position 11780, found in both strains, compared to the republished B6 sequence [23]. This resulted in a T->A transversion, causing an amino acid change of an isoleucine to methionine in the ND5 gene (Table 2). This transversion was found in all three brain mitochondrial populations for both strains and existed in all brain tumor cell lines corresponding to the host strain. This was the only variation found between the B6 strain sequence and the republished B6 sequence [23]. The VM strain also had two additional nucleotide variations compared to the B6 strain. The first variation was found in the ND3 gene at position 9461. This caused a T->C transition, but resulted in no amino acid change. A second variation existed in the poly-A tract of the Arg tRNA, where an adenine was inserted at position 9829 (Table 2). The data show that few mtDNA variations exist between the B6 and VM inbred mouse strains.

### mtDNA somatic mutations in mouse brain tumor cell lines compared to syngeneic host mtDNA sequence

Analysis of the EPEN brain tumor cell line revealed two somatic mutations compared to the syngeneic B6 host strain. One mutation involved an insertion of two adenines in the Arg tRNA at position 9829 (Table 3). This is not expected to alter Arg tRNA function because the mutation occurred in a highly variable site in mouse strains [23]. A second mutation involved a T->C transition in the ND3 gene at position 9461, but did not change the amino acid. Analysis of the CT-2A brain tumor cell line revealed only a single mutation compared to the syngeneic B6 host strain. This involved an insertion of an adenine in the O_L _at position 5182 (Table 3). This mutation is also not likely to have functional consequence [23].

As in the chemically induced EPEN and CT-2A brain tumor cell lines, only a few somatic mutations were found in the VM brain tumor cell lines (VM-NM and VM-M) compared to the syngeneic VM host (Table 3). Two somatic mutations were found in the nonmetastatic VM-NM cell line. The first mutation involved a deletion of an adenine in the O_L _at position 5182. This is the same location where the only variation was found in the CT-2A tumor and is consistent with hypervariability in this region [23]. The second mutation in this cell line involved a T->C transition in the D-loop at position 15584. Neither of these mutations have functional consequence.

**Figure 1:**
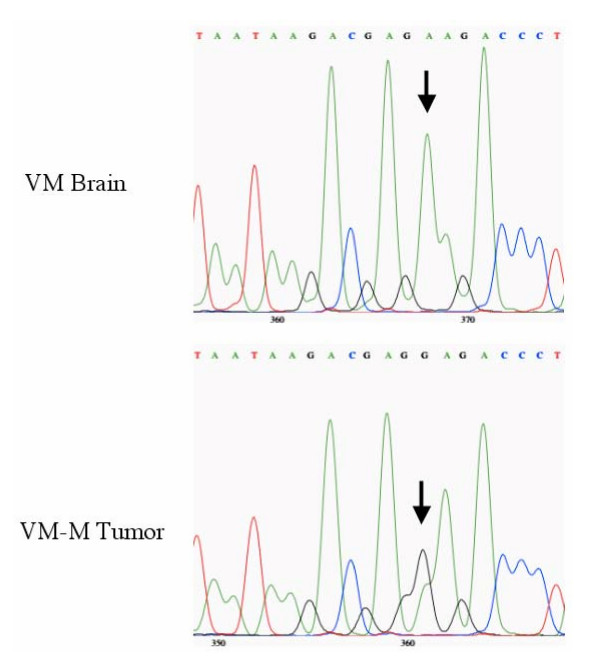
Heteroplasmy in the metastatic VM-M tumor cell line. Mitochondrial 16srRNA revealed a heteroplasmic mutation at position 2159 of an A to G transition. The three VM brain mitochondrial populations (LM, HM, and FM) displayed the same chromotagraph profile. The same level of heteroplasmy was observed in duplicate independent samples for the VM-M cell line. Arrow indicates heteroplasmic nucleotide at position 2159 compared to control.

A single mutation was found in the metastatic VM-M cell line involving an A->G transition in the 16srRNA gene at position 2159. In contrast to the other mtDNA mutations found in either the B6 or the VM tumor cell lines, the A->G transition in the VM-M cell line was heteroplasmic (Figure 1). This heteroplasmic mutation, however, was not found in another independently derived cell line from this metastatic tumor. Viewed together, these data indicate that only a few mtDNA somatic mutations exist in the chemically induced tumors in the B6 mice or in the spontaneous brain tumors in VM mice. Also, most of these mutations were found in hypervariable regions and none are considered pathogenic.

## Discussion

The purpose of this study was to determine if mtDNA mutations existed in chemically induced and spontaneous mouse brain tumors and whether such mutations might provide insight on the development or progression of these experimental brain tumors. To reduce the probability of detecting spurious pathogenic mutations, we compared the mtDNA sequence of each brain tumor cell line with that of normal mtDNA from the syngeneic mouse host strains. Furthermore, to avoid the coamplification of mitochondrial pseudogenes that exist in nuclear DNA, we amplified only large mtDNA fragments (1–1.8 kb in length) in isolated mitochondria [18,57,58]. Because the solid tumors become infiltrated with host stromal cells containing wildtype mitochondria [59], we used cultured tumor cell lines for these studies instead of whole tumor tissue. Hence, our experimental design will detect any mtDNA mutation that could alter mitochondrial function.

Several mtDNA mutations were found in the mouse brain tumor cell lines, but most of these were located in hypervariable mononucleotide hotspot regions, such as in the O_L _and in the Arg tRNA for mouse mtDNA. Moreover, none of these mutations were considered pathogenic since they did not change amino acid sequence and therefore could not alter gene function. Tumor cells in each line were selected from original tumors that may have contained multiple transformed cell types. The lines therefore represent only those cells with the most aggressive malignant growth *in vitro *and *in vivo*. The absence of pathogenic mutations in these tumor cells, further suggests that mtDNA mutations do not likely contribute to the initiation or the progression of these diverse mouse brain tumors.

Most tumors including brain tumors express abnormalities in the number and function of their mitochondria [60,61]. Warburg originally emphasized that the high glycolytic rate of tumors results from diminished or disturbed respiration [24,25]. Later studies in a variety of neural and non-neural tumor systems showed that these respiratory disturbances could involve abnormalities in TCA cycle components, alterations in electron transport, and deficiencies in oxidative phosphorylation [62-66]. Recent studies also suggest that mtDNA mutations may contribute to the respiratory defects in cancer [18]. Our findings indicate that diminished respiration and the glycolytic dependence of the chemically induced CT-2A and EPEN brain tumors or the two spontaneous VM brain tumors studied here do not result from somatic mutations in their mtDNA.

In contrast to previous reports of genetic heterogeneity in normal human brain mtDNA, [40,67] we found no evidence for genetic heterogeneity in mtDNA isolated from synaptic and nonsynaptic mitochondria in the C57BL/6J and VM mouse strains by direct sequencing. On the other hand, our findings that most mtDNA changes in the mouse brain tumors were in hypervariable regions of mononucleotide repeats are in general agreement with previous studies in human brain tumors (Table 4). We detected a single heteroplasmic mutation in the 16srRNA gene of the VM-M metastatic cell line, which was determined by sequence analysis [68]. Since this heteroplasmic mutation was not detected in another independently derived cell line from this tumor, it is unlikely that the mutation is associated with either tumorigenesis or metastasis. The EPEN cell line contained a nucleotide variation compared to the B6 genotype at position 9461 in the ND3 gene. This nucleotide variation in the EPEN cell line also was found in the VM-NM and VM-M cell lines. This polymorphism could be due to a base pair wobble at that location or could result from a nucleotide variation in the original C57BL/6J mouse from which the EPEN tumor arose [44].

The four mouse brain tumor cell lines we examined represent both spontaneous and chemically induced tumors that differ in cell origin, biochemical composition, and angiogenesis. The selection of the cell lines provides a comparison for defining the contribution that mtDNA might play in carcinogenesis. Although we did not find any mtDNA pathogenic mutations in these brain tumors, we do not rule out the possibility that such mutations might occur in other spontaneous mouse brain tumors or in mouse brain tumors induced with other chemical carcinogens. Our findings suggest that mtDNA mutations do not likely contribute to the development or progression of CT-2A or EPEN chemically induced tumors or the metastatic and nonmetastatic spontaneous VM brain tumors.

With the exception of a single variation at nucleotide 11780 in the ND5 gene, our whole mitochondrial genome sequence for the B6 strain was in complete agreement with the recently republished sequence for this mouse strain [23]. This single nucleotide difference involved a T->A transversion that changed isoleucine to methionine. This difference was also found in the VM mouse strain as well as in all examined mouse brain tumor cell lines. It is not yet clear if this variation represents an error in the republished B6 mtDNA sequence or a population genetic variation among B6 mouse strains. Brain mitochondria were fractionated into different populations for genotyping to confirm that there was no mtDNA heterogeneity in hypervariable regions that might be associated with metabolic heterogeneity. Sequencing of all three brain populations also served as an internal control for the genotype of the mouse strains.

In summary, our results show that the mitochondrial genome of these mouse brain tumor cell lines contain mutations in regions of mononucleotide repeats. These mtDNA mutations in the mouse brain tumors are similar to those found previously in hypervariable regions of the D-loop in human brain tumors. However, none of the mouse mutations were pathogenic suggesting that mtDNA changes do not contribute to the carcinogenic progression of these chemically induced and spontaneous mouse brain tumors.

## List of abbreviations

The abbreviations used are: O_L_, origin of replication of the light strand; mtDNA, mitochondrial DNA; kb, kilobase; D-loop, displacement loop; B6, C57BL/6J

## Competing interests

The author(s) declare that they have no competing interests.

## Authors' contributions

MK carried out mitochondrial isolation, amplification and sequencing of brain and tumor mtDNA, sequence analysis and drafted the manuscript. TS participated in the study's design, coordination, and editing of the manuscript.

## Pre-publication history

The pre-publication history for this paper can be accessed here:



## Figures and Tables

**Table 1:** Primers used for amplification of *Mus Musculus* mitochondrial genome

**Primer pair**	**mtDNA bp**	**Forward primer**	**Reverse primer**
1	15309-00040	GGTCTTGTAAACCTGAAATGAAG	GCATTTTCAGTGCTTTGCTTTG
2	16173-01436	CTGATCAATTCTAGTAGTTCC	CAAGCTCGTTAGGCTTTTCAC
3	01314–02880	GAATTACAGCTAGAAACCCCG	CGTATGGACCAACAATGTTAGG
4	02673–03873	GTTATTAGGGTGGCAGAGCCAG	GCCCGATAGCTTAATTAGCTGAC
5	03731–05324	CTTTGATAGAGTAAATTATAGAGG	TAAAATGGCTGAGTAAGCATTAG
6	05194–06593	GTCTTAGTAGAGATTTCTCTACAC	GTTTACTCCTACGAATATGATGG
7	06477–07996	GGAGCAGTGTTTGCTATCATAGC	TGATGGCTACAACGATTGGGAATC
8	07847–09438	GTCTCATCACAAACATTCCCAC	CAGAATCTACTAATTGGAAGTCAG
9	09307–10806	GATTTGAAGCCGCAGCATGATAC	GTCATAGGTGAACTCCATATAATGG
10	10647–12399	GCCCTCATCTTAATCCAAAACC	CTGTAGCTGCGATTAATAGGC
11	12247–13788	CAATCCTCTATAACCGCATCG	GTATTGGGGGTGATTATAGAG
12	13670–15498	CCAACATCATCAACCTCATAC	GTACTAGCTTATATGCTTGGG

The nucleotide positions were based on *Mus Musculus *sequence from Bibb *et al*. 1981 [GenBank: V00711]

**Table 2:** Summary of mtDNA nucleotide variations in C57BL/6J and VM mouse strains^a^

**Mouse strain**	**Nucleotide position**	**Gene**	**DNA change**	**Amino acid change**
C57BL/6J	11780	ND5	T->A	Ile->Met
VM	11780	ND5	T->A	Ile->Met
VM	9829	Arg tRNA	InsA	NA^b^
VM	9461	ND3	T->C	Ile->Ile

Accession numbers for C57BL/6J and VM strains are DQ106412 and DQ106413, respectively

^a ^Nucleotide variation compared to the republished C57BL/6J mtDNA sequence from Bayona-Bafaluy *et al*. 2003

^b ^NA, not applicable

**Table 3:** Summary of mtDNA mutations in experimental mouse brain tumor cell lines^a^

**Brain tumor cell line**	**Metastatic**	**Host genetic background**	**Nucleotide position**	**Gene**	**DNA change**	**State^b^**	**Amino acid change**
EPEN	-	B6	9829	Arg tRNA	InsAA	Homo	NA^c^
			9461	ND3	T->C	Homo	Ile->Ile
CT-2A	-	B6	5182	O_L_	InsA	Homo	NA
VM-NM	-	VM	5182	O_L_	DelA	Homo	NA
			15584	D-loop	T->C	Homo	NA
VM-M	+	VM	2159	16srRNA	A->G	Hetero	NA

^a ^Tumor cell lines mtDNA sequences were compared to host strain brain mtDNA populations

^b ^Homo, homoplasmy; hetero, heteroplasmy

^c ^NA, not applicable

**Table 4:** mtDNA alterations in human brain tumors compared to matched tissue controls

**Type of tumor**	**Number of tumors**	**Number of tumors with mtDNA somatic mutations^a^**	**Number of somatic mutations causing amino acid substitutions^c^**	**Region of mutation**	**Reference**
Neurofibroma	37	23/37	NA^d^	D-loop	Kurtz *et al*. 2004
Glioma	53	20/53	NA	HVRII	Vega *et al*. 2004
Meningioma	11	5/11	NA		
Schwannoma	5	1/5	NA		
Medulloblastoma	15	6/15	2/18	D-loop, Asp tRNA, COXI, CoxII, Thr tRNA, ND4	Wong *et al*. 2003
Gliomatosis cerebri	6	2/6	NA	HVRII	Kirches *et al*. 2003
Glioma	55	12/55	NA	HVRII, D-loop	Kirches *et al*. 2001
Astrocytoma	12	11/12^b^	NA	HVRII	Kirches *et al*. 1999

^a ^Number of tumors with mutations/number of tumors

^b ^2 of the 12 astrocytomas were compared to adjacent brain, the remaining 10 were aligned to Anderson *et al*. 1981 for mutation analysis

^c ^Number of mutations causing amino acid substitutions/total number of mutations found in all tumors

^d ^NA, not applicable because in hypervariable region of the D-loop (HVR)
